# Supplementary material for: Systematic Review of Tumor Segmentation Strategies for Bone Metastases
Source: Cancers (Basel). 2023 Mar 14;15(6):1750. doi: 10.3390/cancers15061750 (PMC10046265; doi:10.3390/cancers15061750)
Supplement: Supplementary file 1 [file cancers-15-01750-s001.zip › cancers-2252961-supplementary.pdf]

	Reference	Technique	Primary Cancer	Image Modality	Dataset	Application	Evaluation Metric	Results	Suitable for Clinical Use or Not	Open-Source Code Availability
	**Deep Learning**									
1	Papandrianos, N., et al., 2020	CNN	Prostate	Bone Scans	586 bone scan images; 368 bone scans of male patients with bone metastasis; 218 bone scans of male patients without bone metastasis	Classification of the bone metastasis	PA<br>Loss<br>Precision<br>Recall<br>F1<br>TPR<br>TNR	**Malignant disease class:**<br>PA: 97.38<br>Loss: 0.087<br>Precision: 0.987<br>Recall: 0.973<br>F1: 0.98<br>TPR: 0.984<br>TNR: 0.95<br><br>**Healthy disease class:**<br>PA: 97.38<br>Loss:<br>Precision: 0.947<br>Recall: 0.975<br>F1: 0.96<br>TPR: 0.945<br>TNR: 0.987	Not addressed	No
2	Chmelik, J., et al., 2018	CNN	Metastases / Not specified the primary cancer	CT	31 patients (13 females, 18 males; mean age 70.8 years; range 46–86 years) involving 626 vertebrae (100 cervical, 371 thoracic, and 155 lumbar)	Segmentation and classification of difficult to define metastatic spinal lesions	TPR<br>TNR<br>AUC<br>YI	**Lytic:**<br>TPR=0.71<br>TNR=0.88<br>AUC=0.8<br>YI=0.59<br><br>**Sclerotic:**<br>TPR=0.74<br>TNR=0.82<br>AUC=0.78<br>YI=0.56	Yes [higher TPR in comparison to the inter-expert agreement. The number of False Positive (FP) detections is still within satisfactory limits (according to the cooperating clinicians)]	No

3	Guo, Y., et al.,2022	CNN with residual connection and hybrid attention mechanism	Lung	Bone Scans	506 patients; 1011 images	Metastases Detection	PA<br>Precision<br>Recall<br>F1<br>AUC	PA: 0.7782<br>Precision: 0.7799<br>Recall: 0.7823<br>F1: 0.7764<br>AUC: 0.8364	Not addressed	No
4	Han, S., J.S. Oh, and J.J. Lee, 2022	CNN (whole body based (WB) and tandem architectures integrating whole body and local patches (GLUE))	Prostate	Bone Scans	5342 patients; 9113 bone scans	Metastases Detection	PA<br>AUC	**GLUE:**<br>PA: 0.9<br>AUC: 0.936–0.955<br><br>**WB:**<br>PA: 0.889<br>AUC: 0.933–0.957, P > 0.05	Potential; doesn't guarantee suitability for clinical practice	No
5	Li, T., et al. 2022	CNN	Lung	Bone Scans	2185 patients	Metastases Detection	PA<br>Precision<br>Recall<br>F1	PA: 0.7392<br>Precision: 0.7592<br>Recall: 0.7242<br>F1: 0.7292	Not addressed	Code available via collaboration agreement by contacting the correspondi ng author
6	Lin, Q., et al., 2020	U-Net and Mask R-CNN	Metastases / Not Specified the primary tumor	CT	76 patients; Age:43 - 87 years	Segmentation of metastasis hotspots	PA<br>CPA<br>Recall<br>IoU	PA: 0.9920<br>CPA: 0.7721<br>Recall: 0.6788<br>IoU: 0.6103	Not addressed	No
7	Moreau, N., et al., 2022	nn-Unet (2 models for	Breast	PET/CT	60 patients; Training: 60 baseline and 104	Metastases Segmentation	DSC<br>Recall<br>Precision	**Baseline:**<br>Mean DSC: 0.66<br>Global DSC: 0.73	Not addressed	No

		baseline and follow-up)			follow-up PET/CT images; Evaluation: 10 patients with one baseline and one follow-up			Recall: 0.72<br>Precision: 0.87<br><br>**Follow-up:**<br>Mean DSC: 0.58<br>Global DSC: 0.64<br>Recall: 0.63<br>Precision: 0.78		
8	Moreau, N., et al., 2020	nn-Unet (2 models for bone and bone lesion segmentation)	Breast	PET/CT	24 patients	Bone and Bone Lesion Segmentation	DSC<br>Recall<br>Precision	**Bone:**<br>Mean DSC: 0.58<br>Global DSC: 0.59<br>Recall: 0.67<br>Precision: 0.82<br><br>**Bone Lesion:**<br>Mean DSC: 0.61<br>Global DSC: 0.61<br>Recall: 0.67<br>Precision: 0.88	Not addressed	No
9	Papandrianos, N., E. Papageorgiou, and A. Anagnostis, 2020	CNN	prostate	Bone Scans	778 whole-body scintigraphy images from 817 different male patients	Identify bone metastasis	PA	PA: 91.42% ± 1.64%	Not addressed	No
10	Song, Y., et al., 2019	Modified Holistically nested Edge Detection, FCN	Metastases /Not specified the primary cancer	CT	21 patients, 250 images	Segmentation of Bone Metastasis	TPR<br>IoU	TPR: 79.8%<br>IoU: 69.2%	Not addressed	No
11	Zhang, J., et al., 2021	U-Net	Metastases /Not specified	Bone Scans	128 original datasets; 2280 sheets of	Segmentation of Bone Metastasis	DSC<br>IoU<br>MAP	DSC: 0.571<br>Mean IoU: 0.633<br>MAP: 0.761	Not addressed	No

			the primary cancer		amplified data volume					
12	Apiparakoon, T., et al., 2020	FPN	Metastases /Not specified the primary cancer	Bone Scans	9824 patients; Whole body scans: Training:680; Validation:200; Testing: 240<br><br>Lesion instance segmentation: chest images: 19,648; labeled:1,088 unlabelled:18,560	Segment abnormal hotspots and classify bone cancer metastases	F1<br>precision<br>TPR<br>TNR	F1: 0.852<br>mean precision: 0.856<br>mean TPR: 0.657<br>TNR: 0.857	Not addressed	No
13	Borrelli, P., et al., 2022	CNN	Lung	PET/CT	320 patients	Segment and quantify tumor burden	CI<br>Hazard Ratio	CI: 95% 1.21–2.21<br>Hazard Ratio: 1.64	Feasible	No, Available upon request from the RECOMIA platform (https:// recomia. org)
14	Chang, C.Y., et al., 2022	CNN	Metastases /Not specified the primary cancer	CT	242 CT scans; 600 images	Detection and Segmentation of lesions	DSC<br>TPR<br>TNR<br>PPV	DSC: 0.83<br>TPR: Global = 95%, local=92%<br>TNR: local = 87%<br>PPV: local = 97%	No	No
15	da Cruz, L.B., et al., 2022	DeepLabv3+ 2.5D, Dual Path	Kidney	CT	300 CT scans; KiTS19 dataset	Tumor Segmentation	PA<br>TPR<br>TNR<br>DSC	PA: 99.71%<br>TPR: 84.24%<br>TNR: 99.82%<br>DSC: 85.17%	Not addressed	No

		Network (DPN)					JI<br>HD<br>ASD	JI: 75.62%<br>HD: 18.39 mm<br>ASD: 3.36 mm		
16	Ding, Y., et al., 2022	V-net	Cervical	CT	130 patients	CTV and OARs Segmentation	DSC<br>JI<br>ASD<br>HD	CTV<br>DSC: 0.85<br>JI: 0.77<br>ASD: 2.58 mm<br>HD: 11.2 mm	Feasible	No
17	Li, L., et al. 2020	3D FCN + fuzzy variational model	Lung	PET/CT	84 Patients; 48 randomly selected CT images were used to train the FCN, and the remaining 36 PET/CT images were used as the test dataset	Tumor Segmentation	DSI<br>TPR<br>PPV<br>Volume error<br>Classification error	DSI: 0.86 ±0.05<br>TPR: 0.86 ±0.07<br>PPV: 0.87 ±0.10<br>volume error: of 0.16 ±0.12<br>classification error: 0.30 ±0.12	Not addressed	No
18	Lu, Y., et al., 2020	U-Net	Lung	PET	25 patients; 1309 PET images; before-radiotherapy (BR) sub-database (769 images); after-radiotherapy (AR) sub database (540 images)	Tumor segmentation	DSC<br>TPR<br>HD<br>JI<br>PPV	**BR:**<br>DSC: (0.8615, 0.7635, 0.6607)<br>TPR: (0.8938, 0.7413, 0.7462)<br>PPV: (0.8990, 0.8050, 0.6223)<br><br>**AR:**<br>DSC: 0.8518<br>TPR: 0.8397<br>PPV: 0.8933	Not Addressed	No
19	Protonotarios, N.E., et al., 2022	U-Net	Lung	PET/CT	87 patients; Lung-PET-CTDx	Tumor segmentation	PA<br>Precision<br>Recall<br>F1	Average PA: 99.332<br>Average Precision: 76.206<br>Average Recall: 57.636<br>Average F1: 60.072	No	No

					open dataset from The Cancer Imaging Archive Database (TCIA); Used only patients who underwent a PET and CT examination with B70f reconstruction kernel		IoU<br>AUC	Average IoU: 49.036		
20	Sartor, H., et al., 2020	CNN	Cervical and Anorectal	CT	266 cases; Training/Validation: Cervical cancer:65; Anorectal cancer: 161;	OAR Segmentation	MSD<br>DSC	**Anorectal cancer:**<br>DSC:<br>– Femoral heads: 0.91–0.92<br>– Bladder: 0.94<br>– Bowel Bag: 0.83<br>MSD:<br>– Femoral heads: 1.93–1.86<br>– Bladder: 2.07<br>– Bowel Bag: 6.80<br>**Cervical cancer:**<br>DSC:<br>– Femoral heads: 0.93–0.94<br>– Bladder: 0.84<br>– Bowel Bag: 0.88<br>– CTVNs: 0.82<br>MSD:<br>– Femoral heads: 1.42–1.49<br>– Bladder: 3.51	Not Addressed	No

								– Bowel Bag: 5.80<br>– CTVNs: 3.89		
21	Tian, H., et al., 2020	FCN	Lung	PET	54 3D PET images; 49 training images and 5 testing images, and then cut them into 2D slices for training. Finally, there was a balanced training set, and the total nearly training number is 14,700	Tumor Segmentation	DSC<br>TPF<br>FPF<br>Precision	DSC: 79.63±7.99<br>TPF: 92.05±5.81<br>FPF: 0.02±0.01<br>Precision: 86.83±7.14	Not Addressed	No
22	Zhang, Y., et al., 2022	GAN	Abdomen, Soft tissue, Liver, Lung, Mediastinum, Bone, Pelvis, Kidney	CT	4427 patients; 10,954 CT scans	Tumor Detection	Mean Pixel Accuracy<br>Precision<br>Recall<br>JI	Mean Pixel Accuracy: 0.9841<br>Precision: 0.9737<br>Recall: 0.9845	Not Addressed	No
23	Zhao, X., et al., 2019	Deep CNN	Lung	PET/CT	84 patients; Training: 84 PET/CT images; Testing: 36 PET/CT images	Tumor Co-segmentation	DSC<br>CE<br>VE	Mean and SD of;<br>DSC: 0.85 (0.08)<br>CE: 0.33 (0.19)<br>VE: 0.15 (0.14)	Not Addressed	No
24	Hu, Q., et al., 2020	mask R-CNN/ K-means /SVM	Lung	CT	13,000 CT images; 39 lung CT exams	Lung segmentation	Position Adjustment<br>Intensity Adjustment	The Mask R-CNN and K-means configuration yielded the best values of Position Adjustment	Not Addressed	No

							Size Adjustment<br>DSC<br>PA<br>TPR<br>Segmentation time	(99.21%), Intensity Adjustment<br>(99.40%), Size Adjustment (97.62%), DSC (97.33%), PA (97.11%) and the second best in TPR (96.58%), after<br>Mask R-CNN and SVM (96.69%)		
25	Lindgren Belal, S., et al., 2019	CNN	Prostate	PET/CT	100 CT scans	Bone Segmentation	Sørensen-Dice index	Sørensen-Dice index: 0.86 (Th7), 0.85 (L3), 0.88 (sacrum), 0.84 (7th rib) and 0.83 (sternum)	No	No
26	Noguchi, S., et al., 2020	U-Net	Lung, Breast and other	CT	In-house dataset: 16 patients; 32 scans; 16218 slices<br>Secondary dataset: 20 patients; 20 scans; 12529 slices<br>Public dataset: 20 patients; 27 scans; 270 slices	Bone Segmentation	DSC	DSC:<br>In-house: 0.983 ± 0.005<br>Secondary: 0.943 ± 0.007<br>Public: 0.947 ± 0.013	Not Addressed	No
27	Xiong, X., et al., 2022	U-Net	Pelvic	PET/CT	34 patients (17 female and 17 male); 34 planning CT scans and 106 FLT<br>PET-CT scans	Pelvis segmentation and uptake quantification	DSC	Average DSC: 0.9396 ± 0.0182	Not Addressed	No
28	Arends, S.R.S., et al., 2022	CNN (two networks for combined	Metastases /Not specified	CT	Training:	Vertebral body segmentation	DSC<br>HD	DSC: 96.7%<br>HD: 3.6 mm	Feasible	No

		approach and sequential approach)	the primary cancer		59 patients (31 female and 28 male); 580 Vertebrae Evaluation: VerSe 2019 dataset; 15 scans; 202 vertebrae			Most robust in external validation: sequential approach (DSC: 94.5% vs 94.4%, $p < 0.001$, HD: 4.5 vs 7.1 mm, $p < 0.001$)		
29	Feng, X., et al., 2020	U-Net	Thoracic	CT	Challenge dataset: 60 thoracic CT scans; Training :36; Offline Testing: 12; Online Testing: 12; Each group has 1:1:1 ratio from the three clinical sites Private Dataset: 45 randomly selected thoracic CT scans; Enhance Network Performance: 30 scans; Final Evaluation: 15 scans	OAR segmentation	DSC MSD HD	**DSC:** SpinalCord : 0.88±0.03 Lung_R : 0.98±0.01 Lung_L :0.98±0.01 Heart :0.92 ± 0.03 Esophagus:0.80 ± 0.04 **MSD:** SpinalCord :0.8±0.2 Lung_R :0.7±0.3 Lung_L : 0.5±0.1 Heart : 2.5 ± 0.8 Esophagus:1.4 ± 0.4 **HD:** SpinalCord :0.8±0.2 Lung_R :0.7±0.3 Lung_L : 0.5±0.1 Heart : 2.5 ± 0.8 Esophagus:1.4 ± 0.4	Feasible to overcoming data heterogeneity in clinical practices with small effort	No
30	Ibragimov, B., et al., 2020	CNN	Liver	CT	122 liver SBRT	Identification of critical regions associated with toxicities after	PA	PA: 73% in terms of the AUROCC. Significantly higher risk scores ($P < 0.05$) of HB toxicity	Feasible	No

						liver stereotactic body radiation therapy		manifestation were associated with irradiation for<br>the hepatobiliary tract in comparison to the risk scores for liver segments I–VIII and portal vein		
31	Lin, X.W., N. Li, and Q. Qi., 2021	U-Net with Residual and Attention Mechanisms	Head and Neck	CT	50 CT images; MICCAI 2019 StructSeg challenge	OAR Segmentation	DSC<br>HD	Average DSC: 79.5%<br>Average HD: 6.99mm	Promising for improving accuracy and efficiency in radiotherapy for clinical doctors.	No
32	Liu, Z.K., et al.,2020	U-Net	Cervical	CT	105 patients; average age ± standard deviation: 51.81 ± 10.14 years	OARs segmentation	DSC<br>HD	**DSC:**<br>bladder :0.924<br>bone marrow: 0.854<br>femoral head left: 0.906<br>femoral head right: 0.900<br>rectum: 0.791<br>small intestine: 0.833<br>spinal cord: 0.827<br><br>**HD:**<br>bladder: 5.098<br>bone marrow: 1.993<br>femoral head left: 1.390<br>femoral head right: 1.435<br>rectum: 5.949<br>small intestine: 5.281<br>spinal cord: 3.269	Not Addressed	No
33	Nemoto, T., et al., 2020	U-net	Lung	CT	232 NSCLC cases; training and validation:	Normal lung segmentation	DSC<br>CI	**DSC:**<br>Smart segmentation: 0.964 [95% (CI), 0.960–0.968]	Need Further Study	No

					200; testing: 32 cases		Wilcoxon signed-rank test (P<0.01)	2D: 0.990 (95% CI, 0.989–0.992)<br>3D U-Net: 0.990 (95% CI, 0.989–0.991)		
34	Nemoto, T., et al., 2020	U-Net	prostate	CT	556 cases; training and validation: 500; testing: 56	Semantic segmentation in radiation therapy planning for prostate cancer	DSC	**Highest DSC:**<br>Prostate:0.85±0.05<br>bladder:0.94 ± 0.04<br>rectum: 0.85 ± 0.07	Not Addressed	No
35	Men, K., J. Dai, and Y. Li, 2017	Deep Dilated CNN	Rectal	CT	278 patients; Training: 218 patients; Testing: 60 patients	CTV and OAR Segmentation	DSC	**DSC:**<br>CTV: 87.7%<br>Bladder: 93.4%<br>Left femoral head: 92.1%<br>Right femoral head: 92.3%<br>Intestine: 65.3%<br>Colon: 61.8%	Not Addressed	No
36	Papandrianos, N., et al., 2020	CNN	breast	Bone Scans	382 patients	Diagnosis of metastatic breast cancer in bones	PA	PA: 92.50%.	Yes, Feasible	No
37	Pi, Y., et al., 2020	CNN	Lung, Pancreatic, Prostate, Thyroid cancer, Breast, Lymphoma, Colorectal, Ureteral carcinoma, Nasopharyngeal-carcinoma,	Bone Scans	13,811 patients; 15,474 annotated examinations; 9595 benign diagnoses;5879 malignant cases; 6699 males (mean age 61.25 ±12.58); 7112 females (Mean age 54.43 ±11.58)	Segment abnormal hotspots and classify bone cancer metastases	F1<br>Precision<br>TPR<br>TNR	F1: 0.852<br>mean precision: 0.856<br>mean TPR: 0.657<br>TNR: 0.857	Feasible for clinical bone scans diagnosis.	No

			Laryngeal carcinoma, Liver, Malignant melanoma, Gastric, Endometrial, Renal, Biliary carcinoma, Bladder, Parotid carcinoma, Esophageal carcinoma, Ovarian carcinoma, Mediastinal malignant tumor, Uterine cervix							
38	Liu, S., et al., 2022	Deep residual CNN (bone scan classification model, a regional segmentation model, an assessment	Metastases /Not Specified the primary cancer	Bone Scans	280 patients with bone metastases; 341 patients with non-bone metastases	Automatically analyze bone metastases	PA<br>TPR<br>TNR<br>DSC	**Classification Model:**<br>PA: 88.62%<br>TPR: 92.59%<br>TNR: 85.51%<br>DSC: 0.7387 (segmentation)<br><br>**Report Generation Model:**<br>PA: 78.05%	Feasible	No

		model for tumor burden and a diagnostic report generation model)								
39	Lou, B., et al., 2019	Multi-task deep NN	Primary (stage IA–IV) or recurrent lung cancer and patients with other cancer types with solitary metastases or oligometastases to the lung	CT	944 patients; internal study cohort: 849 independent validation cohort :95	Generate an image fingerprint that predicts time-to-event treatment outcomes and approximates classical radiomic features	CI<br>C-index<br>Hazard Ratio	C-index: 0·77 (95% CI 0·69–0·92)<br>Hazard Ratio: 3·64 [95% CI 2·19–6·05], p<0·0001)	Yes, pretreatment risk stratification and risk-adapted dose optimization in clinical trials and, in routine clinical practice	No
40	Rao, C., et al., 2021	U-Net	Oropharyngeal	PET/CT	254 patients; Training: 180 patients; Validation: 21 patients; Testing: 53 patients; HECKTOR challenge	Tumor Segmentation	DSC	**DSC:**<br>Validation: 66.9%<br>Test set: 58.7%	Not Addressed	Yes; https://gitlab.com/UM-CDS/projects/image-standardization-and-domain-adaptation/hecktor-

										segmentation-challenge
41	Xue, Z., et al., 2021	CNN	Liver	PET/CT	100 scans	Tumor Segmentation	DSC<br>HD	Global DSC: 71.40 ± 0.45<br>HD: 48.0264 mm	Not Addressed	No
42	Chen, J., et al., 2021	CNN	prostate	SPECT/CT	12 patients; 11 clinical SPECT images; 12 clinical CT images; phantom data	Segmentation for partitioning a quantitative bone single-photon emission CT image into lesion, bone, and background	DSC<br>Recall<br>Precision<br>IoU	**Clinical CT scan:**<br>DSC: 0.794<br>Recall: 0.790<br>Precision: 0.804<br>IoU: 0.661	Further evaluation needed with clinical images	No
43	Hsieh, T.-C., et al., 2021	CNN	Lung, Prostate, Breast, Liver, Nasopharyngeal-carcinoma and other cancer	Bone scans	19,041 patients; Aged 22 to 92 years; 34386 images with metastases; 3041 images without metastases	Metastases Detection	PA<br>Precision<br>Recall<br>F1<br>AUROCC<br>NPV	PA: 0.961<br>Precision: 0.878<br>Recall: 0.599<br>F1: 0.712<br>AUROCC: 0.92<br>NPV: 0.965	Not Addressed	No
44	Biswas, B., S.K. Ghosh, and A. Ghosh, 2020	Pulse-coupled neural network	Brain	CT	250 CT images	Tumor Segmentation	DSC<br>Ratio of Segmentation Error<br>Uniformity Measure<br>JI<br>PPV<br>TPR	Average of:<br>DSC: 32.687<br>Ratio of Segmentation Error: 7.87<br>Uniformity Measure: 0.99<br>JI: 0.96<br>PPV: 0.99<br>TPR: 0.99	Not Addressed	https ://github.com/biswajitcs ecu

45	Yousefirizi, F. and A. Rahmim, 2021	GAN + Mumford-Shah loss functional	Head and Neck	PET/CT	254 patients; Training: 201 patients; Testing: 53 patients; HECKTOR dataset	Tumor and nodal metastases Segmentation	DSC Precision Recall	DSC: 67% Precision: 73% Recall: 72%	Not Addressed	No
Thresholding										
1	Hammes, J., P. Täger, and A. Drzezga, 2018	Hounsfield unit threshold (HUT), SUV threshold (SUVT)	Prostate	PSMA PET/CT	20 68Ga-PSMA PET/CT scans	Automated Quantification of Bone Metastasis	correlation	SUVmax, r^2 5 0.97; SUVmean, r^2 5 0.88; lesion count, r^2 5 0.97	Not Addressed	Yes (https://github.com/jochenhammes/PSMA_Analysis/)
2	Moussallem, M., et al., 2012	Adaptive	Lung	FDG PET/CT	65 lung lesions of 54 patients	tumor segmentation	Mean difference Standard deviation	Lesions greater than 20 mm, (mean difference between measured and calculated data equal to -0.8 ± 9.0%) and an acceptable estimation of CT measurements. For lesions smaller than or equal to 20 mm, the method showed disagreement with the measurements derived from histological or CT data	Yes	No
3	Perk, T., et al., 2018	a statistically optimized regional thresholding	castrate resistant prostate	18F-NaF PET/CT	37 patients	bone lesion detection	TPR TNR	TPR:95.8% TNR: 97.1%	Not Addressed	No

4	Fränzle, A., et al., 2014	simple thresholding/ flood filling algorithm	multiple myeloma	CT	14 CT scans	bone segmentation	PA<br>Centroid difference<br>Mean difference angle	PA: 90%<br>mean centroid difference: 21.7 mm<br>mean difference angle: 1.54◦	Not Addressed	No
5	Nguyen, C.T., et al., 2016	Iterative	Leukemia	PET/CT	17 patients, 51 scans	3D bone marrow segmentation	PA	an average PA of 91.7%<br>worst-case PA of 80.4%	Not Addressed	No
6	Tsujimoto, M., et al., 2018	Fixed	Not specified	SPECT/CT	35 patients; (17 males, 18 females); mean age was 61 ± 14 (range 30–80) years	segmentation and detection of increased uptake regions in Bone Scans	TPR<br>Correlation coefficient	TPR: 71%<br>Correlation coefficient 0.868	Not Addressed	No
Clustering/Classification										
1	Chu, G., et al., 2014	Random Forest	prostate	Bone Scans	213 scans training set of 140 subjects and a testing set of 73 subjects	Bone Tumor Segmentation	JI	JI: 0.57±0.27	Not Addressed	No
2	Elfarra, F.-G., M.A. Calin, and S.V. Parasca, 2019	Parallelepiped Classification	Not specified	Bone Scans	12 patients; mean age ± standard deviation: 61.08 ± 12.48 years	Metastases Detection	classification accuracy<br>κ coefficient	overall classification accuracy: 87.58 ± 2.25%<br>κ coefficient: 0.8367 ± 0.0252	Not Addressed	No
3	Hinzpeter, R., et al., 2022	Gradient-boosted tree	Prostate	CT	67 patients; mean age 71 ± 7 years; range: 55–84 years	Invisible Bone Metastases Detection	PA<br>CI<br>TPR<br>TNR	PA: 85%<br>CI: 0.76–0.92, p < .001<br>TPR: 78%<br>TNR: 93%	Not Addressed	No
4	Slattery, A., 2017	fuzzy clustering	Lymphoma	PET–CT	248 patients	delineate regions of interest within all phantoms	dSMAD<br>JI<br>HD<br>Δx	Mean dSMAD: 0.454<br>Mean JI: 0.979<br>Mean HD: 38.5<br>Mean Δx: 7	Yes	No

5	Wiese, T., et al., 2011	SVM	Breast, Lung, prostate	CT	10 patients with sclerotic metastatic disease in the spine; 8 patients (80%) with prostate cancer, one patient (10%) with lung cancer, and one patient (10%) with breast cancer; 50-75 years	Cancer Metastasis Detection	TPR<br>CI	TPR:77.4%<br>CI: (0.71, 0.84)	Not Addressed	No
6	AbuBaker, A. and Y. Ghadi, 2020	Wavelet transform and SVM	Lung	CT	60 CT images	Cancer Detection	TPR<br>FPR	TPR: 94.5%<br>FPR: 7 cluster/image	Not Addressed	No
7	Hussain, L., et al., 2019	Naïve Bayes, DT and SVM	Lung	CT	76 patients; 954 images i.e., 568 from SCLC subjects and 377 from NSCLC subjects	Cancer Detection	TPR<br>TNR PPV<br>NPV<br>TA<br>FPR<br>AUC	The highest detection accuracy was obtained with (TA=100%) with entropy, SIFT and texture features using Naïve Bayes, texture features using SVM Polynomial. Moreover, the highest separation was obtained using entropy, morphological, SIFT and texture features with (AUC=1.00) using Naïve Bayes classifier and texture features using Decision tree and SVM polynomial kernel	Not Addressed	No

8	Markel, D., et al., 2013	Decision Tree / K-nearest neighbors	Lung	18-FDG PET/CT	34 cases; both small cell and non-small cell lung carcinoma (stages T1–T4);	Segment GTV	TPR<br>DSC<br>TNR	TPR: 73.9%<br>DSC: 0.607<br>TNR: 99.2%	Not addressed	No
9	Naqiuddin, M., et al., 2019	Fuzzy C-Mean (FCM)	Brain tumor	CT	97 slices; 1 Male and 1 Female Patients aged 52 and 72 respectively	Lesion demarcation to study the characteristics of brain tumor cases of CT scan image so that the information of grey level intensity and Hounsfield Units can be obtained	Gray Level Intensities Hounsfield Units	**Gray Level Intensities:**<br>Tumor: 64.5<br>Boundary (Lesion): 92<br>Skull: 254.5<br>Cerebrospinal fluid: 80.5<br>Gray Matter: 87.5<br>White Matter: 126<br><br>**HU:**<br>Tumor: 10<br>Boundary (Lesion): 47.5<br>Skull: 3000<br>Cerebrospinal fluid: 8.5<br>Gray Matter: 39<br>White Matter: 30	Not addressed	No
10	Sarker, P., et al.,2017	K-means clustering	Lung	CT	70 patients; 22489 CT images	Lung Tumor Segmentation and Classification	PA<br>TPR<br>TNR	PA: 95.68%<br>TPR: 86.4%<br>TNR: 98%	Not addressed	No
11	Polan, D.F., S.L. Brady, and R.A. Kaufman, 2016	Random Forest algorithm	neck–chest–abdomen–pelvis	CT	21 patient images sections, Additional 100 randomly selected patients	Tissue Segmentation	DSC<br>TPR<br>TNR<br>PA	**For 21 patients:**<br>DSC: 0.86 ± 0.03<br>**For 100 patients:**<br>TPR: 0.91 (range: 0.82–0.98),<br>TNR 0.89 (range: 0.70–0.98),<br>PA 0.90 (range: 0.76–0.98)	Not addressed	No

12	Zhou, H., et al., 2018	SVM	Lung	CT	348 patients	Diagnosis of Distant Metastasis	PA<br>AUC	PA: 71.02%<br>AUC: 72.84%	Not addressed	No
13	Zhang, J., et al., 2020	SVM	Lung	PET/CT	82 patients	Solitary Pulmonary Nodules Classification	PA (95% CI)<br>TPR (95% CI)<br>TNR (95% CI)<br>PPV (95% CI)<br>NPV (95% CI)<br>AUC (95% CI)	PA (95% CI): 0.85 (0.838–0.862)<br>TPR (95% CI): 0.889 (0.684–1)<br>TNR (95% CI): 0.818 (0.59–1)<br>PPV (95% CI): 0.8 (0.552–1)<br>NPV (95% CI): 0.9 (0.714–1)<br>AUC (95% CI): 0.854 (0.637–1)	Not addressed	No
Statistical										
1	Rachmawati, E., et al., 2020	Active Shape Model	Metastases /Not specified the primary cancer	Bone Scan	19 bone scan images, 200 landmark points in total	Cancer Metastasis Detection	minimum cumulative error	minimum cumulative error: 0.0446	Not addressed	No
2	Guo, Y., et al., 2014	Fuzzy Markov Random Field Model	Lung	PET/CT	7 patients;	Tumor Segmentation	DSC	DSC: 0.85 ± 0.013	Not Addressed	No
3	Martínez, F., et al., 2014	Geometrical shape model	Pelvic	CT	116 patients; Training: 30 patients; Testing: 86 patients	Organ Segmentation	DSC	**DSC:**<br>Prostate: 0.91<br>Bladder: 0.94<br>Rectum: 0.89	Useful as a support in final delineation	No
4	Ninomiya, K., et al., 2018	Bayesian delineation framework / anatomical-	Prostate	CT	44 patients; median age: 72 year; range: 52-87	Bayesian delineation framework of CTVs for prostate	Average location error<br>DSC	Average location errors of CTV PAs along the anterior-posterior and superior-	Yes	No

		features-based machine learning (AF-ML) / Probabilistic atlases			year; stage: T1-T4, N0, M0	to address the drawbacks of unknown CTV locations		inferior directions without AF-ML were 5.7±4.6 mm and 5.5±4.3 mm, respectively, whereas the errors along the two directions with ANN, which showed the best performance, were 2.4±1.7 mm and 2.2±2.2 mm, respectively. The average DSC between reference and estimated CTVs for 44 test cases were 0.81±0.062 with ANN		
Atlas based approaches										
1	Kim, N., et al., 2020	Atlas-based automatic segmentation	Endometrial and Cervical	CT	75 patients	Contouring in patients with endometrial and cervical cancers	DSC<br>HD	best results / 60 patients: (DSC, 0.79; HD, 19.7 mm)<br>worst results/ 20 patients (DSC, 0.75; p = 0.012; HD, 21.3 mm; p = 0.002)	Yes	No
2	Hanaoka, S., et al., 2017	Landmark-guided diffeomorphic demons' algorithm	Bone disease	CT	20 CT volumes in spine dataset and 50 whole torso CT datasets	Segmentation of the whole spine and pelvis in CT images	mean distance error<br>DSC	mean distance error: 0.59 ± 0.14 mm<br>DSC: 0.90 ± 0.02	Not Addressed	No
3	Ruiz-España, S., et al., 2017	Probabilistic atlas	Metastases / Not specified the primary tumor	CT	21 patients; 11 male and 10 female (58.47 ± 13.78 years, mean ± standard deviation	Segmentation of the Spine with a Special Focus on Ribs Suppression	DSC<br>HD<br>MSD	HD: 15.51 ± 2.74 mm,<br>DSC: 91.01 ± 3.18 %<br>MSD: 0.66 ± 0.25 mm	Not Addressed	No
4	Yusufaly, T., et al., 2020	Multi-atlas Approach	Cervical	FDG-PET/CT	144 patients	Active Bone Marrow Sparing	DSC<br>CI	DSC: 0.73<br>CI: 95%	Yes. Tested in multi-institutional trial, NRG-GY006	No

						Radiation Therapy				
5	Fritscher, K.D., et al., 2014	Label fusion/ Multi atlas-based segmentation	head and neck	CT	18 CT images	segmentation of head-neck CT	DSC<br>mean absolute distance<br>max HD between the auto segmentation results and expert segmentations	**average DSC:**<br>right parotid gland: 0.81<br>left parotid gland: 0.84<br>brainstem: 0.86	Yes	No
Region based approaches										
1	Yang, B., et al., 2018	Multi-scale template matching and region growing	Lung	PET/CT	5 patients; 10 3D PET images	Tumor segmentation	DSC<br>TPR<br>FPR	DSC: 86.91±5.83%<br>TPR: 5.78±7.34%<br>FPR: 0.033±0.003%	Not Addressed	No
2	Dong, R., et al., 2017	Connected component Labeled Graph Cuts Algorithm	Metastases /Not specified the primary tumor	CT	100 CT slices	Vertebral Segmentation	TPR<br>FPR	TPR: 96.72%<br>FPR: 1.84%	Not Addressed	No
3	Sato, S., et al., 2018	salient region feature registration	Metastases /Not specified the primary tumor	CT	Synthetic data; No further info	Enhance the bone metastasis from CT images	TP<br>FP	rotating reference image at rotation angle<br>theta alpha = -10 degrees:<br>TP 100.0 % and FP 12.16 %.<br>Gaussian filter to rotate image:<br>TP 70.40 % and FP 0.00 %.	Not Addressed	No

								artificial pseudo lesion region to rotated image: TP 99.45 % and FP 17.89 %. adding random noise of 5% to rotated image: TP 83.05 % and FP 16.95 %.		
4	Elsayed, O., et al., 2015	Region Growing	Lung	CT	200 patients; each with 150 to 500 images,	Pulmonary Nodules Detection	PA	PA: 98%	Not Addressed	No

Sensitivity: TPR; specificity: TNR; Area Under Curve: AUC; Youden's Index: YI; False Positive: FP; Accuracy: PA; Class Pixel Accuracy: CPA; Intersection Over union: IOU; F1-score: F1; Dice Similarity Coefficient: DSC; Hausdorff Distance: HD; Jaccard Index: JI; Positive Predictive Value: PPV; Confidence Interval: CI; Mean Surface Distance: MSD; Dose Volumetric Histograms: DVH; True Positive Fraction: TPR; False positive Fraction: FPF; Classification Error: CE; Volume Error: VE; Mean Absolute error: MAE; Mean Error: ME; Mean Median 75% percentile 95% percentile 100% Percentile of symmetric mean absolute distance: dSMAD; Euler characteristic: Δ¬x; positive Predictive Value: PPV; Negative Predictive Value: NPV; Total Accuracy: TA; False Positive Rate: FPR; Similarity Index: SI; Average Surface Distance: ASD; average dice similarity indexes: DSI; area under the receiving operator characteristic Curve: AUROCC
